# Twelve tips for teaching medical students online under COVID-19

**DOI:** 10.1080/10872981.2020.1854066

**Published:** 2020-12-07

**Authors:** Zhehan Jiang, Hongbin Wu, Huaqin Cheng, Weimin Wang, A’Na Xie, Sarah Rose Fitzgerald

**Affiliations:** a Institute of Medical Education, Peking University, Beijing, China; b National Center for Health Professions Education Development, Peking University, Beijing, China; c Peking University Health Science Center, Peking University, Beijing, China; d University of Massachusetts, Amherst, Massachusetts, USA

**Keywords:** COVID-19, medical MOOC, e-learning, SPOC, assessment

## Abstract

Universities worldwide are pausing in an attempt to contain COVID-19’s spread. In February 2019, universities in China took the lead, cancelling all in-person classes and switching to virtual classrooms, with a wave of other institutes globally following suit. The shift to online platform poses serious challenges to medical education so that understanding best practices shared by pilot institutes may help medical educators improve teaching. Provide 12 tips to highlight strategies intended to help on-site medical classes moving completely online under the pandemic. We collected ‘best practices’ reports from 40 medical schools in China that were submitted to the National Centre for Health Professions Education Development. Experts’ review-to-summary cycle was used to finalize the best practices in teaching medical students online that can benefit peer institutions most, under the unprecedented circumstances of the COVID-19 outbreak. The 12 tips presented offer-specific strategies to optimize teaching medical students online under COVID-19, specifically highlighting the tech-based pedagogy, counselling, motivation, and ethics, as well as the assessment and modification. Learning experiences shared by pilot medical schools and customized properly are instructive to ensure a successful transition to e-learning.

## Introduction

COVID-19 presents the education community with an unprecedented challenge for three reasons: 1) the virus’ severe contagion and mortality risk factors, 2) daily changes in guidance from state and country authorities, and 3) the lack of knowledge of the virus. To help mitigate the impact of the virus on the community, schools have taken steps to limit the virus’ spread by reducing the density of the population on campus. According to UNESCO, 46 countries on five different continents announced school closures to contain the spread of COVID-19 as of 12 March, 2020. This practice has led to a call for rapid transition from on-site educational settings to completely web-based environments, which have imposed great challenges for schools and teachers, especially those lacking the relevant experience and facilities. Recognizing the pandemic circumstances, [1, provided five tips for universities forcing instructors to turn to remote teaching: (1) don’t convert the entire lecture to video, 2) don’t merely rely on live video, 3) encourage students’ engagement and feedback, 4) communicate with students often, and 5) focus more on struggling students. These, however, are aiming at institutions offering general college education, which can be very different from medical school teaching.

Medical education is driven by imparting to persons seeking to become physicians the knowledge and skills required for the prevention and treatment of disease [2]. Medical education involves highly interdisciplinary courses, clinical experience, and rapid and ongoing knowledge updates so that the schools are expected to take up the challenge of modifying their curricula to prepare students for better responding to the current and future trends that stem from ‘population health maintenance and the consequent practice’ in the field [3]. Expressly, most medical schools set students in physical settings for 1–3 years where their knowledge foundations are built; students’ physical presence in both inpatient and outpatient settings has been a successful practice of early clinical immersion experiences and the clerkship curriculum. The second half of medical school education requires students to participate in clinical rotations, sub-internships, and/or research projects.

**Figure 1.**
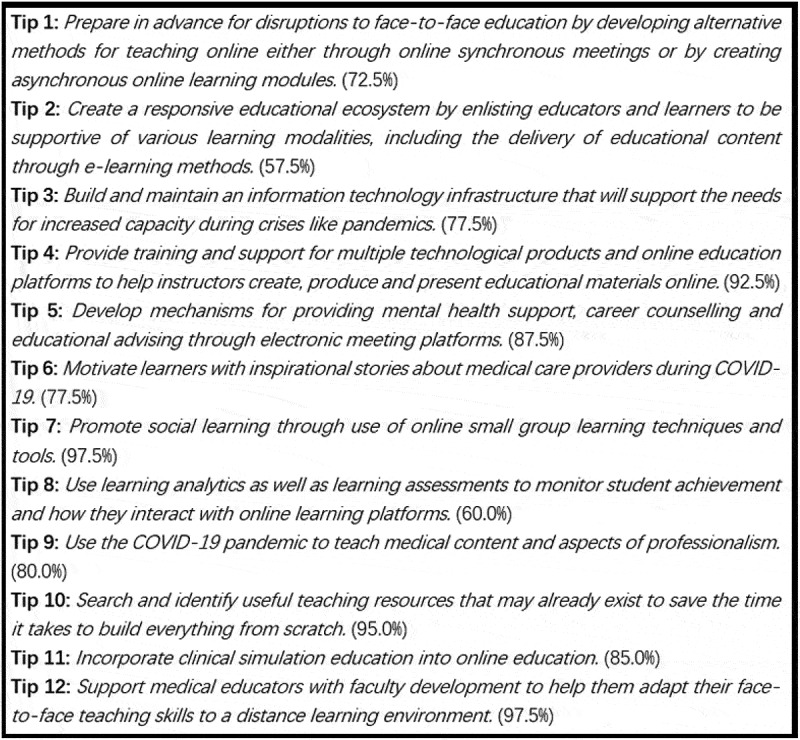
All Twelve Tips for Teaching Medical Students Online under COVID-19

To accommodate changes in education during COVID-19, most schools, including those specializing in medical teaching, have implemented a social distancing rule to suspend onsite education activities. This practice has been recognized as one of the most effective preventative strategies since the emergence of COVID-19, pending development of a vaccine and/or treatment [4]. As one of the earliest responding countries, China urged campus shutdowns in mid-February, 2020 under the Chinese Ministry of Education (MOE) initiative ‘Disrupted classes, undisrupted Learning’ [5], and the policy remains active up to the time this paper was written. According to the Chinese MOE, as of 4 April 2020, 1,454 higher education institutions were operating online, with around 7.133 million online sessions (i.e., 94,200 courses) involving more than 95,000 instructors and 1.18 billion students. Correspondingly, the National Centre for Health Professions Education Development (NCHPED) in the country surveyed all affiliated medical schools to submit ‘best practices’ reports after having switched to online mode [6]. These reports present great value, primarily in the form of references or memos, for medical schools moving on-site education to online teaching or improving hurriedly constructed online instruction plans. The tips below are organized in a ‘zooming-in’ flow that spans from school-level to instructor-level practice. Details covered in the tips may be overlapping to a small degree; however, each tip reveals a unique perspective aiming to help medical schools differently. These tips are listed in Figure 1, where the percentages show the proportions of collected reports voting over the tips.

## Tip 1: prepare in advance for disruptions to face-to-face education by developing alternative methods for teaching online either through online synchronous meetings or by creating asynchronous online learning modules

Similar to other institutions in the education sector, most medical schools could not foresee the spread of the virus and therefore did not have sufficient time to prepare for online teaching on a large scale in such a short period. For those schools having weaker e-learning experience and/or infrastructure, the obstacles can increase exponentially. As a result, it is evitable to encounter unexpected difficulties in both online instruction and institutional policy during the transition. Strategies should therefore be devised to enable one to pivot from face-to-face to synchronous-at-a-distance to asynchronous learning environments; specifically, strategies are needed to allow shifting from in-person lectures to video conference lectures to recorded lectures methods of teaching. Accordingly, to the ‘best practices’ reports, instructors and school administrators admitted that, even with successful examples set by peer institutions and colleagues, education-related decisions and teaching activities could deviate from theoretical expectations. It’s unreasonable to assume that once a decision is made or a teaching framework is set to face-to-face approaches, one should stick with the pre-determined way. (1) Developing alternative methods for teaching online either through online synchronous meetings and/or (2) creating asynchronous online learning modules not only refer to the class equipment, network security, familiarity with the operation, lesson content planning but also refers to embracing online classes from a teacher’s heart and standing in the class perspective to make adequate preparation. Good practices include determining the software platforms used for different functions and defining engagement code to regulaate students’ activities online.

## Tip 2: create a responsive educational ecosystem by enlisting educators and learners to be supportive of various learning modalities, including the delivery of educational content through e-learning methods

The rapid transition to a complete e-learning environment demands more efficient reactions to organize teaching activities. This corresponds to the concept of flexible learning, which is defined as ‘a set of educational approaches and systems concerned with providing learners with increased choice, convenience, and personalization to suit their needs. In particular, flexible learning provides learners with choices about where, when, and how learning occurs, by using a range of technologies to support the teaching and learning process.’ [7]. Among many stakeholders, flexible learning is seen as the ‘mission of instructors.’ Applying online learning to provide flexible education, however, is difficult and will be impossible to realize, if the instructors are the only responsible party. Accordingly, flexible learning should be a widespread and systematic effort. Flexible learning goes far beyond singular improvements like pedagogy or technology enhancement [8,9,10], for example, schools need to ensure that faculty, staff, and students receive/use the right equipment, software, and training to be able to interact at a distance. Unsurprisingly, in the ‘best practices’ reports, most schools mention a call for encouraging all related parties to work, contribute, and compromise (if necessary) together to create a responsive ecosystem in a short period. For instance, graduate studies departments/offices allow students ready for finishing their academic programs to defend online. Librarians offer expanded hours and diverse modes of reference services to help both teachers and students, academic affairs staff allocate aid to poverty-stricken students. Administrators work more closely with assessment teams to supervise the quality of the various efforts to help smooth the transition. It’s also suggested that early adopters of technology should take the lead in the community, such that the multiplier effect benefiting a faster ecosystem construction can be achieved.

## Tip 3: build and maintain an information technology infrastructure that will support the needs for increased capacity during crises like pandemics

The network facility defines how successful e-learning implementation can be. In response to COVID-19, medical schools have quickly transitioned the entire workforce to online formats that include not only teaching activities, but also administration, admission, finance, and human resources. The ‘best practices’ reports show that network congestions or crashes rank top among the issues and problems found during the transition. This phenomenon reiterates the fact that many schools did not have adequate capacity of both servers and IT services to handle the sudden network loads. Some suggestions were made to assist both reducing the facility burdens and smoothing the e-learning deployment process: (1) optimizing shunts for website visiting behaviours such that the server won’t be overloaded, (2) monitoring statistics and server parameters more often to ensure quick fixes when problems occur, (3) encouraging instructors to use reliable and mature platforms and applications (e.g., Zoom and Skype) to avoid consuming schools’ on-site network capacity, 4) reminding instructors and students to ensure the availability of backup options or alternatives such that they can still be connected when the original teaching channels fail, 5) renting or purchasing cloud services (preferably those with fast deployment and reliable features such as Azure, Amazon, and Alibaba products) to expand current network capacity [11,12], 6) establishing a catalogue of relevant systems with links to training and support regarding the network issues, and other similar approaches. Mixing both internal and external resources and optimizing the work assignments proportionally can yield an effective solution bundle to establish a stable environment for e-learning during COVID-19. Further, attention and support should be given to parties (e.g., instructors and students) having difficulties in accessing the Internet. For instance, rural areas may have unstable network connections and students in poverty may need hardware for participating in classes. Administrators must therefore identify the population in need of hardware support and provide necessary help to ensure equipment that is needed in e-learning is accessible.

## Tip 4: provide training and support for multiple technological products and online education platforms to help instructors create, produce and present educational materials online

The network facility defines how successful e-learning implementation can be. In response to COVID-19, medical schools have quickly transitioned the entire workforce to online formats that include not only teaching activities, but also administration, admission, finance, and human resources. The ‘best practices’ reports show that network congestions or crashes rank top among the issues and problems found during the transition. This phenomenon reiterates the fact that many schools did not have an adequate capacity of both servers and IT services to handle the sudden network loads. Some suggestions were made to assist both reducing the facility burdens and smoothing the e-learning deployment process: (1) optimizing shunts for website visiting behaviours such that the server won’t be overloaded, (2) monitoring statistics and server parameters more often to ensure quick fixes when problems occur, (3) encouraging instructors to use reliable and mature platforms and applications (e.g., Zoom, Skype, and DingTalk) to avoid consuming schools’ on-site network capacity, 4) reminding instructors and students to ensure the availability of backup options or alternatives such that they can still be connected when the original teaching channels fail, 5) renting or purchasing cloud services (preferably those with fast deployment and reliable features such as Azure, Amazon, and Alibaba products) to expand current network capacity [11,12], 6) establishing a catalogue of relevant systems with links to training and support regarding the network issues, and other similar approaches. Mixing both internal and external resources and optimizing the work assignments proportionally can yield a useful solution bundle to establish a stable environment for e-learning during COVID-19. On the other hand, attention and support should be given to parties (e.g., instructors and students) having difficulties in accessing the Internet. For instance, rural areas may have unstable network connections, and students in poverty may need hardware for participating in classes. Administrators must therefore identify the population in need of hardware support and provide necessary help to ensure equipment that is needed in e-learning is accessible.

## Tip 5: develop mechanisms for providing mental health support, career counselling and educational advising through electronic meeting platforms

Health care professionals have shown a boost of anxiety during the pandemic [13–15]. The fact that the treatment periods could last weeks and months and the spread isn’t likely to be appropriately controlled in a short period requires health care professionals be able to perform to their full potential over an extended time interval. Meanwhile, due to the lack of personal protective equipment (e.g., ventilators) and protections (e.g., masks, goggles, and coveralls), health care professionals encounter more significant exposure risks, extreme overburdens, moral dilemmas, and a rapidly changing practice environment such that their anxiety levels are dramatically increased [16–18]. Timely mental health help is considered to be essential to support health care professionals, especially for those working directly with COVID-19 patients [19]. Expected to be future health care professionals, students in medical schools also experience similar anxiety [20,21]. This anxiety is amplified by media reporting on the increasing number of deaths of both patients and medical staff, which includes school instructors who are called to the frontline. Studies have indicated that overwhelming anxiety can deflate students’ academic performance [22]. Therefore, easing students’ occupational anxiety about their future career is critical to prevent the downward effect of emotional and mental disruption. The ‘best practices’ reports show that medical schools adopt multiple ways to help maintain students’ mental health during COVID-19: 1) providing online psychological counselling sessions (preferably by licensed counsellors) to students by telephone and online chatting, 2) requiring students to report their anxiety and stress levels frequently, 3) recommending tips and resources related to mental health and social distancing, 4) encouraging online social activities (e.g., online book clubs and talk-radio call-in programs), and other methods of this kind.

## Tip 6: motivate learners with inspirational stories about medical care providers during COVID-19

In a medical school setting, the majority of the students possess autonomous motivation [23], which includes intellectual curiosity, professional autonomy, altruism, and interest in human relationships [24]. These motivation components correspond well to the two-factor theory [also known as Herzberg’s motivation-hygiene theory and dual-factor theory; 25], which states that there are factors in the job-motivating employees that can result in improving their job satisfaction and career expectation. In the present context, learning how the efforts of health care professionals help and save lives provides significant career inspiration. The ‘best practices’ reports show that all medical schools have promoted outstanding deeds of medical staff, especially those who are instructors in school and called to the frontline, among students. Without disrupting working schedules, many schools also invited those working in the frontline to share their stories and encourage students to 1) work hard on learning knowledge and 2) keep a strong sense of mission to better contribute to society in the future. Besides, some schools have established new seminar series cultivating professionalism in medical students, via reporting physicians and nurses’ work against COVID-19; ideas such as ‘having as a professional is not just a desirable condition, but also a requirement to safeguard patient safety and improve patient care outcomes’ were emphasized so that students can better understand the role of health care professionals: being held responsible for their own professional performance, and also for upholding the trustworthiness of the whole medical profession. According to the ‘best practices’ reports, with the support of evidence collected from schools’ surveys, students reported a substantial growth in motivation, which can positively affect their e-learning process.

## Tip 7: promote social learning through use of online small group learning techniques and tools

Students’ social presence, the ability to perceive others in an online environment, has been shown to influence their motivation, engagement, and retention of courses [26]. In addition, attention should be paid to online interaction and timely feedback, as learning occurs through knowledge sharing among students and peer interaction. Studies have found that online communication and discussion can improve the quality of students’ e-learning; Students can be influenced by the active participation of their peers and feedback can increase the interaction between teachers and students [27]. In particular, a flipped classroom design has been used in many schools, according to the ‘best practices’ reports, to enhance the pre- and post-class discussions among students via chat apps, emails, and other educational management information systems. On the other hand, instructors should work as panellists to navigate students’ interactions in some scenarios (e.g., streaming videos before an instant discussion is initiated). Specifically, correcting off-track discussions and providing appropriate directions are needed when the interactions demand instructors intervene. Previous research identified group size in asynchronous online discussions influence students’ perception of social presence; smaller groups are more amenable to building interpersonal relationships with other participants and help members come together on common goals [28]. Specifically, when placed in small permanent discussion groups, the students perceived the online learning context to (a) be more sociable, (b) have a more positive atmosphere, and (c) afford group cohesion more easily [29]. Students also noted that in smaller groups, they felt closer to each other, whereas, during whole-class discussions, it was hard for them to keep track of all the posts and develop impressions of their classmates.

## Tip 8: use learning analytics as well as learning assessments to monitor student achievement and how they interact with online learning platforms

**Figure 2.**
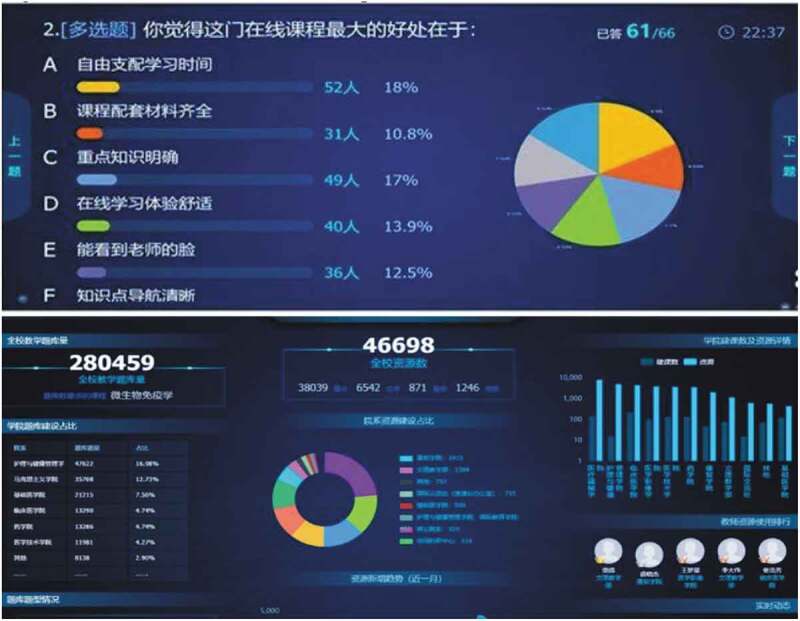
Two Data Visualization Examples

This tip involves surveying, online tracking, sampling and other data science approaches for evaluation purposes. However, above all, defining learning objectives should be the driving factor of the series of assessments in which the students will show learning progression [30]. In general, these objectives are used to navigate formative and summative frameworks to form a complete assessment system for a school [31]. In this particular time, however, formative assessment should be emphasized as it provides more responsive feedback focused on quality improvement and professional development rather than grades or ranking. Examples of formative assessment of learning objectives may include websites, discussion forums and online discussion spaces, real-time online chat, and communication apps. The ubiquitous availability of video and audio on audio devices has enabled performance feedback and assessment in authentic contexts for clinical and simulated learning environments. Ideally, these assessments should be based on high-quality evidence and theory-informed assessment and evaluation strategies [32,33]. Data science approaches have played an essential role in the assessments, in which analytics are built to monitor student engagement and progress toward learning goals. According to the ‘best practices’ reports, most medical schools have deployed visualization panels to track students’ engagements, assignment completions, feedback, and other relevant indicators. Figure 2 provides two data visualization cases: the upper panel shows survey results collected from students such that the instructor can learn the pros and cons of the assigned course, and the lower panel presents key statistics (e.g., the time length of the online sessions across majors) for administrators to assess the progress of the transition.

## Tip 9: use the COVID-19 pandemic to teach medical content and aspects of professionalism

**Figure 3.**
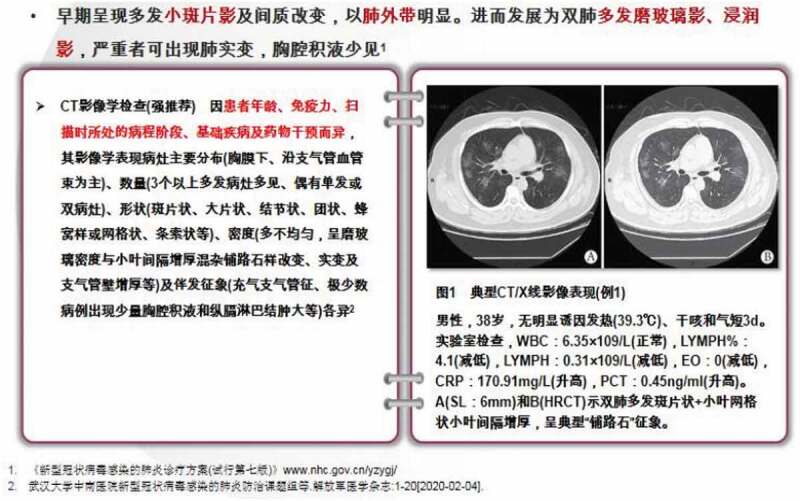
COVID-19 Cases Used in Teaching Materials

The massive exposures to COVID-19 news, the match between the pandemic background and the students’ professional area, and the rapid knowledge updates/research about the virus among medical schools and institutions have created convincing incentives for raising learner awareness of medical students [34]. Given the high level of learner awareness, integrating COVID-19-related knowledge into learning routines under the current circumstance will be particularly effective. The ‘best practices’ reports show that most instructors have embedded relevant content into their teaching cases. As Figure 3 illustrates, a COVID-19 patient’s chest CT was used in a medical imaging class, with the support of the instructor’s notes and slides. Other examples include (1) using results of COVID-19 antibody tests as examples in physical health assessment classes and (2) addressing potential COVID-19 cure development in vaccine-related classes. Li and colleagues also recommended embedding (1) taking a COVID-19-related history and physical examination, (2) decision-making for triage, (3) proper escorting of suspected patients, and (4) self-protection and avoiding contamination into teaching routines and simulation training (2020). In addition to referring to COVID-19 cases in existing teaching frameworks, some medical schools also established new courses and/or seminars, such as ‘Health Emergency Management’ and ‘Epidemiology’ corresponding to the outbreak. For those classes with research components or biomedical/bioinformatic courses, openly accessible COVID-19 data, such as the centralised repository of individual-level information on patients with laboratory confirmation [35], can be used in teaching demonstrations or assignments.

## Tip 10: search and identify useful teaching resources that may already exist to save the time it takes to build everything from scratch

A viable way of successfully transiting to e-learning is integrating existing content from schools themselves, peer institutions, or other Massive Open Online Course (MOOC) and Small Private Online Course (SPOC) platforms [36,37,38]. 39, claimed that probing and locating high-quality educational resources among the thousands of educational resources that are published, although difficult, can be beneficial to both instructors and students, especially from the perspective of cost-effectiveness. Service providers such as edX and Udacity have well-established courses so that instructors can use the ready-for-use lectures, materials, and/or assignments. In terms of medical education resources, NCHPED presents a list containing 22 channels and websites made of more than 2,700 medical courses, where most of them are free to public. Other less-known resources deserve careful credibility evaluations. As the ‘best practices’ reports indicate, instructors did admit that searching appropriate teaching resources, especially for those who have been rooted in the traditional on-site mode for a long time, is not easy, despite awareness of online resource availability. One of the challenges is the fact that most solid content is presented in English sets obstacles for instructors and students whose English language skills are not adequate. Instructors need to consider finding resources in their native languages or preparing lessons further in advance so that materials in non-native languages can be translated properly. Meanwhile, class sizes, students’ mastery statuses, clinical training, and other factors should be taken into consideration, when the instructors are selecting teaching materials and creating lesson plans. Further, openly accessible e-books have been largely adopted to eliminate the need for procurements of physical copies- a traditional way of distributing required textbooks to students in China. Last but not least, communicating with curriculum design specialists (or staff with similar titles) can help instructors better embed and tailor existing resources to their courses.

## Tip 11: incorporate clinical simulation education into online education

**Figure 4.**
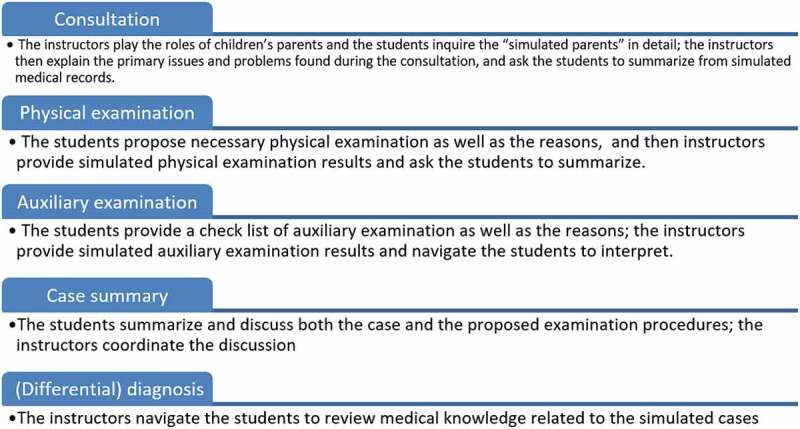
A Standardized Patients Example from a Paediatrics Department

**Figure 5.**
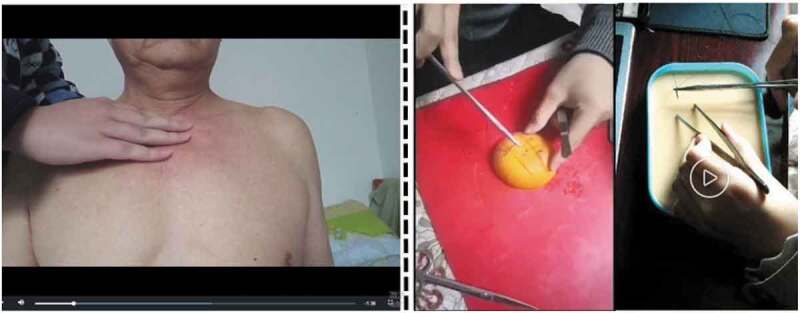
Videos Recording Completing Process of Homework Assignments

Mandated residency training policy in many countries has emphasized the importance of clinical simulation training more than any time in medical education history. In response to COVID-19, clinical skill sessions may occur online or, in some cases, may be deferred [40]. What’s more, many countries have provided guidelines suggesting that medical schools pause students’ clinical rotations which makes virtual clinical simulation a viable, if not the only, way of replacing of the actual clerkship [41]. However, under the social distancing circumstances, medical schools have tried deploying various approaches with limited availability, according to the ‘best practices’ reports. These approaches can be classified as (1) a flipped classroom design, (2) online competence by design, (3) online problem-based learning, and (4) any combinations of the previous approaches [44]. To illustrate, teaching practice via standardized patients (SP) from a paediatrics department was conducted in Figure 4. In addition to the aforementioned example, other simulation approaches include (1) consultation exercises by phone, (2) uploading videos of completing SP-based assignments and hands-on practice on substitute items as seen in the right and the left panels of Figure 5, respectively. Regarding case studies, software and simulation repositories have been used to support problem-based learning, where instructors can provide supports according to students’ performance. Further, quality assurance should be emphasized, continuous efforts should ask for institutional support collaboration, and experience should be shared across institutions to enhance the virtual simulation training. All these simulation approaches, however, should be carefully differentiated and properly used in accordance with studying stages; instructors must evaluate the appropriateness of the available programs and make the decision that matches the students’ and/or classes’ mastery levels (e.g., pre-clerkship or clerkship would entail different simulation approaches).

## Tip 12: support medical educators with faculty development to help them adapt their face-to-face teaching skills to a distance-learning environment

A large proportion of instructors were not confident in a completely online learning environment, due to lack of experience according to the ‘best practices’ reports. To fill this need, all surveyed schools provided specialized support to direct instructors to the pool of available tech options. Distributing instructional videos and workflows as well as calling for individualized assistance have been widely adopted. Pedagogical guidelines and tips are not limited to technology applications only, but also the proper ways of choosing the suitable ones, fusing teaching plans and methods into them, delivering knowledge effectively with flexibility, and collecting feedback for further adjustments. These guidelines correspond to the call for a catalogue of all systems linked with training and support. On the other hand, objectivist modes (i.e., knowledge transferring from teachers) would be preferred when students need to be informed in a limited time and constructivist modes (i.e., knowledge construction) demand more advanced knowledge levels of the students, and more qualitative feedback from the teachers [45]. Effective communication has been recognized as good medical practice [11,46], which should entail its education components given the present context. Indeed, effective communication among instructors has proved to be beneficial for improving pedagogy [47]. The forms of the communication include peer reviews, case discussion, sharing sessions, demonstration classes, co-teaching, and guest speaker invitation. The point is treating the teacher community as an asset to learn from and help each other [48], especially when an instructor encounters pedagogical difficulties in scenarios such as simulation learning activities. Finally, several schools announced that special attention was paid to young/new instructors, as a result, mentoring by senior instructors and other similar supports were created and/or emphasized. Although the support comes in different forms, planning its stages matters to work efficiency, for example, direct assistance, skill-directed training, and community building need to be achieved in order.

## Conclusion

Because of inadequate preparation and continued struggles against COVID-19, medical schools around the world face the challenges of carrying forward teaching activities completely online. Medical education institutions in China were the earliest entities closing campuses and have both successful and unsuccessful experience summarized in their ‘best practices’ reports which were submitted to NCHPED. This paper presents 12 tips extracted from the reports aiming to assist other medical schools to achieve a smooth transition to quality e-learning within a short period of time. Although the tips by no means cover all elements granting a successful transition, they can serve as reference for schools that demand essential knowledge in teaching modes, infrastructure construction, platform integrations, and other educational policy decisions.
